# Investigation on Non-Isothermal Crystallization Kinetics of Polyethylene Terephthalate-Polyethylene Naphthalate Blends

**DOI:** 10.3390/polym17212893

**Published:** 2025-10-29

**Authors:** Qianqian Liang, Kexin Wang, Yong Jiang, Guilin Li, Feng Yang, Ya Cao, Ming Xiang

**Affiliations:** 1 State Key Laboratory of Polymer Materials Engineering, Polymer Research Institute, Sichuan University, Chengdu 610065, China; 2 National Insulation Material Engineering Technical Research Center, Mianyang 621000, China

**Keywords:** polyethylene terephthalate, polyethylene naphthalate, crystallization kinetics, cold crystallization

## Abstract

This study aimed to solve two problems of polyethylene terephthalate (PET) films, namely, their slow crystallization rate and insufficient thermal stability, by using polyethylene naphthalate (PEN) as a modifier to prepare PET-PEN blends with varying PEN contents (0%, 0.9%, 1.8%, and 9%). Fourier-transform infrared spectroscopy (FTIR), differential scanning calorimetry (DSC), and other methods were used to systematically investigate the effects of the PEN content and cooling rate (5–40 °C/min) on the non-isothermal crystallization behavior and kinetics of the blends. The results indicate that PET and PEN exhibit excellent compatibility. As the PEN content increases, the glass transition temperature (*T_g_*) of the blend increases, while the melting point (*T_m_*) and relative crystallinity decrease. PEN exerts an effect on the crystallization temperature (*T_c_*)—“heterogeneous nucleation—diffusion control—steric hindrance effect”. The cold crystallization behavior depends on the PEN content and cooling rate. Samples with PEN content did not exhibit cold crystallization at low cooling rates. The observed non-isothermal crystallization kinetics show that PEN transforms the growth dimension of PET crystals from three-dimensional to two-dimensional, significantly reducing the absolute values of the crystallization rate constant (*Z_c_*) and crystallization activation energy (Δ*E*). Δ*E* tends to stabilize when the PEN content reaches or exceeds 1.8%. In summary, PEN achieves precise control of PET non-isothermal crystallization through the mechanism of “heterogeneous nucleation—diffusion control—steric hindrance effect”. The research results provide theoretical support for the optimization of processing technology for PET-PEN blend films in high-end fields such as food packaging and electronic insulation.

## 1. Introduction

Polyethylene terephthalate (PET) is a semi-crystalline thermoplastic polyester produced by a condensation reaction between terephthalic acid (PTA) and ethylene glycol (EG) [1]. Because of its excellent mechanical strength, chemical stability, transparency, and processability [2], PET has become one of the most widely used polyester varieties in the field of polymer materials [3]. PET film is widely used in key applications, such as food packaging, flexible electronic substrates, optical films, and industrial insulation films, due to its high tensile strength, good barrier properties, and low cost [4]. It is currently the most widely produced and applied polyester film variety in the world.

However, the practical application of PET film faces significant challenges, which limit its expansion in high-end scenarios [5]. First, during the melting, processing, and long-term use of PET, low-molecular-weight oligomers (mainly cyclic trimers) generated by the degradation of molecular chains are prone to migrate towards the film surface and precipitate [4]. This not only poses the risk of contaminating the contents in food packaging but also causes the corrosion of device pins and decreased insulation performance in electronic applications [6]. Second, the crystallization behavior and physical properties of PET have inherent deficiencies. Its slow crystallization rate results in low film processing efficiency. In addition, PET has poor low-temperature toughness and a low thermal deformation temperature, making it prone to brittle cracking or deformation when used in packaging transported at low temperatures and in high-temperature automotive environments [7].

To address these issues, researchers have conducted extensive research on PET modification, which can be divided into two categories: chemical modification and physical modification [8]. Chemical modification involves adjusting the regularity of PET molecular chains by introducing a third monomer (e.g., isophthalic acid or cyclohexanedimethanol) for copolymerization. For instance, research has shown that the crystallization rate of cyclohexanedimethanol-modified PET (PETG) is much lower than that of traditional PET, and although amorphous transparent films can be prepared, their thermal stability decreases accordingly [9,10,11]. Surface grafting modification, such as plasma-induced grafting of hydrophilic groups, can reduce surface precipitates and improve pollution resistance. However, the process is complex, and the modified layer is prone to detachment. In terms of physical modification, inorganic nanoparticle fillers (such as TiO_2_, SiO_2_, and graphene) [12,13] are commonly used [14,15]. However, nanoparticle aggregation can lead to a decrease in film transparency. Organic polymer blending has become the most common approach to PET modification due to its ease of operation and flexible performance control [16]. Previous studies [17,18] have attempted to blend PET with polybutylene adipate terephthalate (PBAT) to improve toughness, but the low heat resistance of PBAT can lead to a decrease in the thermal deformation temperature of the blend. Blending with polycarbonate (PC) enhances heat resistance, but PC and PET have limited compatibility, which requires the addition of compatibilizers [19].

Polyethylene naphthalate (PEN), a polyester material with a similar structure to PET [20], is an ideal choice for high-performance modification of PET [21]. PEN is formed by the condensation of 2,6-naphthalenedioic acid (NDA) and ethylene glycol (EG) [22], and the rigid conjugated structure of the naphthalene ring in the molecular chain gives it significant performance advantages over PET [23].

PEN, a polymer similar to PET, has a high glass transition temperature (*T_g_* ≈ 120 °C) and a heat deformation temperature range of 120–130 °C, which are over 40 °C higher than those of PET [24,25]. PEN also has a barrier rate for oxygen and water vapor that is 3–5 times higher than that of PET and has excellent weather resistance [26]. Moreover, under long-term ultraviolet irradiation, the performance degradation rate is only 1/3 that of PET [27]. In terms of mechanical properties, the rigidity of the naphthalene ring endows PEN with a higher tensile strength and elastic modulus, while its low-temperature toughness is superior to that of PET [28]. More importantly, both PEN and PET contain ester-based (-COO-) structures in their molecular chains, with good thermodynamic compatibility [25]. During the blending process, a homogeneous system can be formed without additional compatibilizers, effectively avoiding the phase separation problems that occur in other blending systems.

Regarding the PET/PEN blend, some basic research has been conducted. Early studies have shown that the addition of PEN can significantly improve the thermal stability of PET [29]. In terms of crystallization behavior, some scholars have found that during the isothermal crystallization process, the naphthalene ring in the PEN molecular chain can serve as a heterogeneous nucleating agent for PET, promoting PET crystal nucleation and increasing the crystallization rate by 1–2 times. Murakami et al. [30] prepared PEN/PET and investigated the mechanical properties and structure formation of unoriented amorphous blend films. They claimed that, above 100 °C, pure PET films were unstretchable due to partial melting and a low stretching rate, while the blend films were stretchable. Jabarin et al. [31] analyzed PET/PEN blends’ crystal structures and morphologies via WAXS, SAXS, and density measurements. For quiescently crystallized blends, the results indicated independent crystallization; for strain-induced crystallized blends, WAXS analysis showed that lattice parameters changed with composition, and density measurements revealed that density decreased as PEN content increased compared with homopolymers. Maurizio Fermeglia et al. [32] carried out mesoscale molecular simulations to model and predict the behavior of PET/PEN blends. Different simulations were performed to study and compare pure homopolymer blends with blends characterized by the presence of PET/PEN block copolymers acting as a compatibilizer. The authors found that many-scale modeling seemed to successfully estimate PET/PEN blend properties and behavior with varying concentrations and processing conditions. H. Wagner et al. [33] prepared blends of PET and PEN and then processed them into biaxially drawn films. Samples taken from the bi-oriented films were then investigated by performing dynamic rheology experiments in the melt state. The results indicate that despite the effect of transesterification reactions, the investigated PET/PEN blend systems consisted of a microseparated phase of PEN platelets in a PET matrix. M Tsuji et al. [34] prepared uniaxially oriented thin films of PET, PEN, and their blends by applying shear strain to their respective melts, and they studied the resulting morphologies using transmission electron microscopy. They found that in PET/PEN blends, the morphologies changed from the PET type to the PEN type as PEN content increased.

However, previous research has predominantly focused on the isothermal crystallization process (constant crystallization temperature), which differs greatly from the non-isothermal crystallization that occurs in actual processing conditions. The crystallization kinetics (such as changes in crystallization temperature, crystallization rate, and crystallization activation energy with PEN content) of PET/PEN blends under non-isothermal conditions have not yet been studied systematically, and the regulatory effect of PEN on the PET crystallization mechanism requires further clarification.

The non-isothermal crystallization process is more consistent with actual PET processing technology, and its kinetic parameters directly determine the production efficiency and final performance of the film. Based on this, this study used PET as the matrix to prepare PET-PEN blends with different PEN contents. DSC was applied to systematically study the effect of PEN content on the non-isothermal crystallization kinetics and behavior of the blends, revealing the regulatory effect of PEN on the non-isothermal crystallization rate, crystallization activation energy, and crystal structure of PET. The aim is to provide theoretical support for the optimization of PET-PEN blend film processing technology and the development of high-performance films.

## 2. Experimental

### 2.1. Materials

**Scheme 1:**
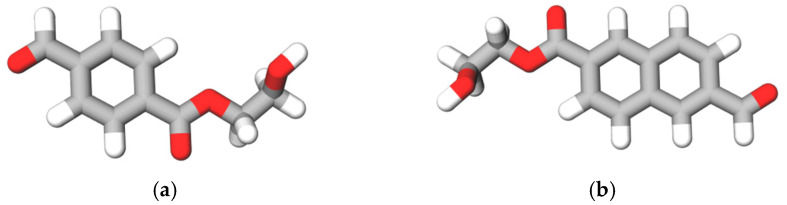
Repeating units of (**a**) PET and (**b**) PEN.

The PETN masterbatch (PET:PEN = 91:9) (tradename PETN, Dongcai New Materials Co., Ltd., Mianyang, China), PET (with the same molecular structure as PET in the PETN masterbatch; produced by Jiangsu Yizheng Chemical Fiber Co., Ltd., Yizheng, China), phenol, trichloromethane, 1,1,2,2-tetrachloroethane, potassium hydroxide, and ethanol, all of analytical grade, were purchased from Shanghai McLean Biochemical Technology Co., Ltd., Shanghai, China. The illustration of repeating units of PET and PEN were shown in Scheme 1.

### 2.2. Sample Preparation

#### 2.2.1. Raw Material Pretreatment

Due to the strong hygroscopicity of PET and PETN, moisture during the melting process can easily hydrolyze and break their molecular chains, leading to a decrease in intrinsic viscosity and an increase in carboxyl end-group content. Therefore, vacuum drying pretreatment is required: PET or PETN was place into a vacuum drying oven (DZF-6020, Shanghai Yiheng Scientific Instrument Co., Ltd., Shanghai, China) and dried for 6 h at 120 ± 5 °C and vacuum degree ≤ −0.095 MPa until the moisture content was below 50 ppm (detected using a Karl Fischer moisture analyzer, C30S, Mettler Toledo International Trade (Shanghai) Co., Ltd., Shanghai, China). After cooling to room temperature, the samples were sealed and stored to avoid secondary moisture absorption [35,36].

#### 2.2.2. Blending Ratio Design and Premixing

According to the target PEN content (0 wt%, 0.9 wt%, 1.8 wt%, 9 wt%), the amounts of raw materials were calculated to ensure that the actual PEN content in the blend system deviates from the design value by ≤0.05 wt%. The specific ratio is shown in Table 1.

PET and PETN blends with the above ratios were added separately to a high-speed mixer (SHR-10A, Zhangjiagang Yunfan Machinery Co., Ltd., Zhangjiagang, China) and then mixed at a speed of 200 rpm and room temperature for 10 min to ensure uniform dispersion of raw material particles and avoid local PEN agglomeration during subsequent extrusion processes. Then, the mixture was immediately transferred to a sealed bag to prevent moisture absorption.

#### 2.2.3. Twin-Screw Extrusion Blending Granulation

The PET/PETN mixture raw materials were blended, extruded, and pelletized using the TE-35 co-rotating twin-screw extruder (screw aspect ratio L/D = 36:1) from Nanjing Keya Machinery Co., Ltd., Nanjing, China. The extrusion process parameters were set as follows: the temperature of the feed section (zone 1) was 190 ± 5 °C to preheat and prevent material bridging, the temperature of the compression section (zones 2–3) was 240 ± 5 °C to achieve preliminary melting and eliminate a small amount of volatile matter, the temperature of the melting section (zones 4–5) was 260 ± 5 °C to ensure complete melting and forced mixing of the raw materials, the temperature of the homogenization section (zones 6–7) was 255 ± 5 °C to further homogenize the melt and reduce shear heat, and the temperature of the head (zone 8) was 265 ± 5 °C to stabilize the melt pressure and ensure uniform extrusion. The screw speed was 80 rpm, and the vacuum degree was set to ≤−0.09 MPa to remove small-molecule volatiles generated during the melting process. After extrusion, the molten material strip was extruded and immediately entered the circulating water cooling tank (water temperature 25 ± 2 °C) for rapid cooling to room temperature. Then, it was pulled by a traction machine to the granulator and cut into cylindrical PET-PEN pellets.

#### 2.2.4. Drying After Granulation and Preparation of Test Samples

Blended pellets are prone to absorbing environmental moisture during cutting and storage and require secondary drying for subsequent performance testing. The blended pellets were placed in a vacuum drying oven (DZF-6020, Shanghai Yiheng Scientific Instrument Co., Ltd., Shanghai, China) and dried for 3 h at 90 ± 5 °C and a vacuum degree below −0.095 MPa until the moisture content was ≤30 ppm (Karl Fischer method detection). The pellets were then cooled to room temperature for later use. All test samples must be stored in a dryer and characterized and analyzed within 24 h.

### 2.3. Intrinsic Viscosity Test

An intrinsic viscosity test was carried out according to “Testing methods of fiber grade polyester (PET) chip” (GB/T 14190-2017) published by the Standardization Administration of China in 2017 [37]. Phenol and tetrachloroethylene were mixed thoroughly in a 1:1 mass ratio to form a solvent. The sample was thoroughly dissolved in the prepared phenol tetrachloroethylene solvent at 120 °C, with a solution concentration of 0.5 g/mL. The intrinsic viscosity measurement was performed using a Ubbelohde viscometer at 25 ± 0.05 °C. The value of [*η*] was calculated using the following equations [38]:

(1) $$\eta_{r}=\frac{t_{1}}{t_{0}}$$

(2) $$\eta_{\mathrm{sp}}=\frac{t_{1}-t_{0}}{t_{0}}=\eta_{r}-1$$

(3) $$[\eta ]=\frac{\sqrt{1+1.4\eta_{sp}}-1}{0.7c}$$

where *ŋ*_r_ is the relative viscosity, *ŋ*_sp_ is the specific viscosity, *t*_1_ is the time for the solution to flow through the Ubbelohde viscometer (s), *t*_0_ is the time for the solvent to flow through (s), and *c* is the solution concentration (0.5 g/dL).

### 2.4. Carboxyl End-Group Test

The Carboxyl End-Group test was carried out according to “Testing methods of fiber grade polyester (PET) chip” (GB/T 14190-2017) published by the Standardization Administration of China in 2017 [37]. Phenol and trichloromethane were mixed in a ratio of 2:3 (by volume), and 2 g of the sample was then placed in a ground triangular flask. After that, 50 mL of the mixed reagent was added for dissolution. After complete dissolution, 5–4 drops of bromoblue indicator were added, and then the solution was titrated with a standardized potassium hydroxide ethanolic solution (c(KOH) = 0.015 mol/L). A blank experiment was conducted under the same conditions. The carboxyl end-group content was calculated according to Equation (4):

(4) $$X\;=\frac{(V\;-\;V_{0})c\;\times {\;10}^{-3}}{m}$$

In the equation, X represents the carboxyl group content of the sample, mol/t; V represents the volume of the titration solution consumed by the sample, mL; V_0_ represents the volume of the titration solution consumed by the solvent, mL; c represents the concentration of the potassium hydroxide ethanol standard solution, mol/L; and m represents the quality of the sample, g.

### 2.5. Diethylene Glycol Test

The sample was degraded in ethanolamine under high-temperature conditions, and diethylene glycol content in the filtrate was detected using gas chromatography.

### 2.6. Fourier-Transform Infrared Spectroscopy (FTIR)

A Frontier Fourier-Transform Infrared Spectrometer (Thermo Scientific Nicolet iS5, Thermo Fisher Corp, Waltham, MA, USA) was used, with a resolution of 4 cm^−1^, ATR attachment, wavenumber range of 4000 to 400 cm^−1^, DTGS detector, and 16 scans.

### 2.7. Differential Scanning Calorimetry (DSC)

DSC measurement was performed on a Mettler Toledo DSC 3+ (Metter Toledo Corp., Zurich, Switzerland). The measurement was performed under a nitrogen flow rate of 50 mL/min using 5–8 mg of the sample.

For the non-isothermal crystallization kinetics test, the thermal protocol was as follows [39,40]: The sample was first heated from 25 °C to 280 °C at a heating rate of 30 °C/min and maintained at 280 °C for 4 min. Then, it was cooled from 280 °C to 25 °C at different cooling rates (5, 10, 20, and 40 °C/min, respectively) and maintained at 25 °C for 4 min, and the cooling crystallization curve of the sample was recorded. Finally, it was heated again from 25 °C to 280 °C at a heating rate of 20 °C/min, and the heating curve of the sample was again recorded.

## 3. Results and Discussion

This study focuses on PET-PEN blends with different PEN contents (0%, 0.9%, 1.8%, 9%) and systematically explores the regulation of PEN on the chemical structure, crystal morphology, thermal stability, and non-isothermal crystallization kinetics of the blends through characterization methods such as Fourier-transform infrared spectroscopy (FTIR) and differential scanning calorimetry (DSC), providing theoretical support for the optimization of PET-PEN blend film processing technology and high-performance applications.

### 3.1. Preliminary Characterization of Sample Molecular Structure

Firstly, the intrinsic viscosity, carboxyl end-group content, and diethylene glycol content of the samples were studied. The results obtained are shown in Table 2.

In Table 2, the intrinsic viscosity reflects the average molecular weight and entanglement degree of polymer chains and is a key indicator for evaluating the integrity of molecular chains. According to the data in Table 2, the intrinsic viscosity of PET is 0.798 dL/g, indicating the intact state of unmodified PET molecular chains; After adding PEN, the intrinsic viscosity of the blend decreased, with PEN-0.9% (0.601 dL/g) and PEN-9% (0.588 dL/g) showing a more significant decrease; the intrinsic viscosity of PEN-1.8% (0.671 dL/g) slightly increased, but it was still lower than that of PET. In general, the intrinsic viscosity of the blend is within a reasonable range, and there is no severe degradation, ensuring that the differences in subsequent crystallization behavior are due to the effect of PEN rather than excessive molecular chain breakage. The content of carboxyl end groups reflects the degree of degradation and terminal activity of the molecular chain, and an increase in content usually indicates hydrolysis or thermal oxidative degradation of the molecular chain. The results in Table 2 indicate that the carboxyl end-group content of PET is 11.9 mol/t. The carboxyl end-group content of PEN-0.9% sharply increases to 20.7 mol/t, while that of PEN-1.8% drops to 15.4 mol/t, and that of PEN-9% further decreases to 9.9 mol/t (lower than PET). Diethylene glycol (DEG) is a byproduct of PET synthesis, and its content directly affects the regularity of molecular chains, which may interfere with crystallization behavior. The diglycol content in the PET sample is 0.63 wt%, which is similar to that in the PEN-0.9% (0.73 wt%), PEN-1.8% (0.60 wt%), and PEN-9% (0.60 wt%) blends, without significant fluctuations.

### 3.2. FTIR

**Figure 1:**
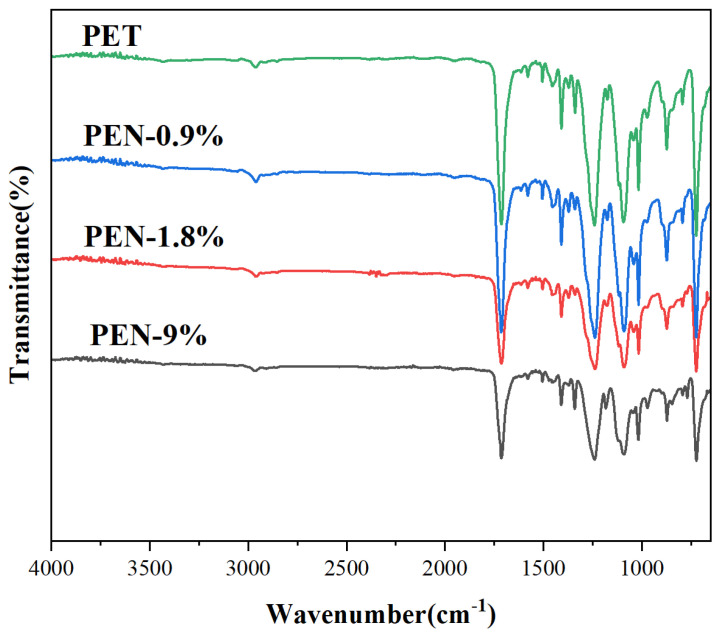
FTIR spectrum of PET and PET-PEN blends.

The FTIR spectra of the samples in Figure 1 showed that all blended samples retained the characteristic functional group absorption peaks of polyester materials: the ester group C=O stretching vibration peak at 1725–1728 cm^−1^, the C-O-C asymmetric stretching vibration peak at 1240–1248 cm^−1^, and the methylene group C-H stretching vibration peaks at 2920 cm^−1^ and 2850 cm^−1^. PEN is primarily recognized from the exclusive vibration peak of the naphthalene ring (around 800 cm^−1^, while the PET benzene ring peak is located at 730 cm^−1^, without overlap). It can be seen that with the increase in PEN content, the peak intensity at 800 cm^−1^ gradually increases and the peak shape is symmetrical, without broadening or shoulder peak phenomenon, indicating that PEN is uniformly dispersed in the PET matrix and no local agglomeration occurs.

No new functional group peaks appeared, confirming that the blending was a physical mixing process that did not damage the polyester main chain structure of PET or PEN and ruling out the potential interference of chemical reactions with crystallization behavior. Ultimately, the results confirm good compatibility between PET and PEN in the PET-PEN blend system, providing a molecular basis for PEN to uniformly regulate PET crystallization.

### 3.3. DSC Measurement

**Figure 2:**
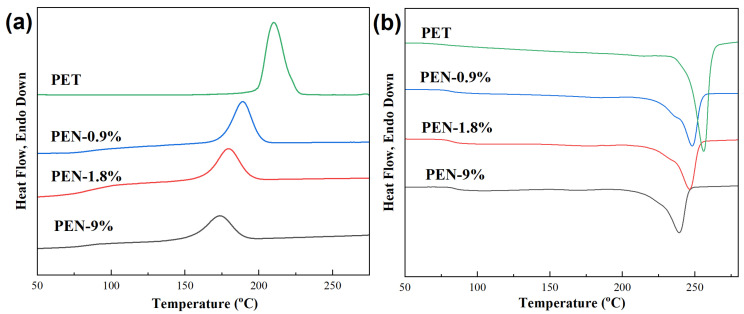
DSC (**a**) cooling curves and (**b**) subsequent heating curves of the samples after eliminating previous thermal history. The cooling rate and subsequent heating rate were both 10 °C/min.

The crystallization and melting behaviors of the samples were tested using DSC, and the obtained DSC cooling curves and subsequent heating curves are shown in Figure 2. The glass transition temperature *T_g_*, crystallization peak temperature *T_c_*, melting point *T_m_*, and relative crystallinity *X_c_* of the samples were obtained from the graph, and the results are listed in Table 3.

The crystallinity (*X_c_*) was calculated using the following equation:

(5) $$X_{c}^{\mathrm{blend}}=\frac{∆H_{m}^{\mathrm{blend}}}{w\;(\mathrm{PET})\times ∆H_{f}\;(\mathrm{PET})+w\;(\mathrm{PEN})\times ∆H_{f\;}\left(\mathrm{PEN}\right)}\times 100\%$$

where $∆H_{m}^{\mathrm{blend}}$ is the experimental melting enthalpy obtained from DSC, and w(PET) and w(PEN), respectively, represent the mass fractions of PET and PEN components. Δ*H_f_* (PET) = 117.6 J/g and Δ*H_f_* (PEN) = 103 J/g are the enthalpies of fusion of perfect crystalline PET [41] and PEN [42].

The crystallization curves in Figure 2a demonstrate that with the increase in PEN content, the crystallization temperature *T_c_* of the sample significantly decreases, from 210.0 °C for PET to 173.6 °C for PEN-9%, indicating that the addition of PEN significantly reduced the crystallization temperature of PET. The changes in the glass transition temperature *T_g_*, melting point *T_m_*, and relative crystallinity of the samples can be obtained from the temperature rise melting curve in Figure 2b. It can be seen that all samples exhibit a single *T_g_*, indicating good compatibility between PEN and PET. As the PEN content increases from 0% to 9%, *T_g_* increases from 80.9 °C to 84.5 °C. This might be due to the steric hindrance of the naphthalene ring on PEN [43], which limits the movement of PET segments and leads to an increase in *T_g_*. At the same time, the melting point *T_m_* decreased from 256.8 °C to 239.2 °C, and crystallinity decreased from 49.4% to 25.5%, indicating that PEN significantly inhibited the integrity of PET crystallization.

### 3.4. Non-Isothermal Crystallization Kinetics

**Figure 3:**
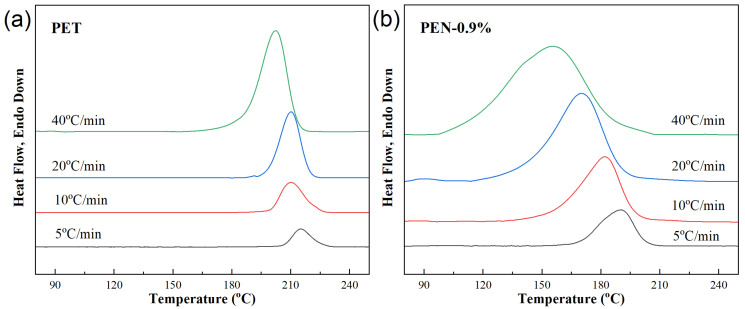

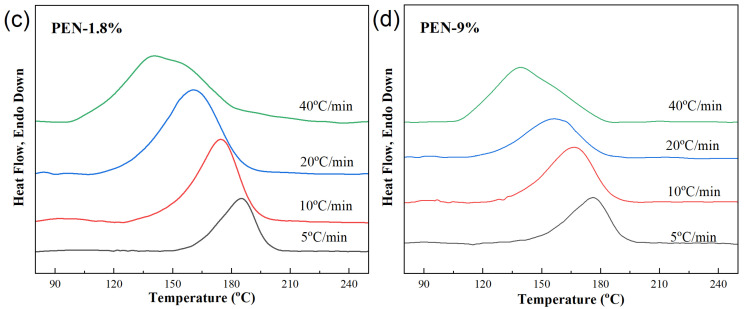
DSC cooling curves of samples at different cooling rates. (**a**) PET, (**b**) PEN-0.9%, (**c**) PEN-1.8% and (**d**) PEN-9%.

Figure 3 shows the DSC crystallization curves of different samples at different cooling rates (5, 10, 20, 40 °C/min). The non-isothermal crystallization parameters of PET-PEN blends at different cooling rates were obtained from Figure 3, including crystallization starting temperature *T*_0_, crystallization peak temperature *T_c_*, crystallization ending temperature *T_e_*, crystallization enthalpy Δ*H_c_*, and half crystallization time *t*_1/2_, which are listed in Table 4.

The crystallization behavior of all samples (including PET) shows a consistent trend with increasing cooling rate: the higher the cooling rate, the more the crystallization heat release peak shifts towards lower temperatures, and the wider the peak shape. This is because the larger the cooling rate *φ*, the slower the migration of molecular segments and the slower the formation of the lattice, and the corresponding relaxation time increases. Therefore, the formation of crystal nuclei and the growth of crystals are limited, resulting in a wider range of crystallization temperatures and larger hysteresis [44].

Taking pure PET as an example, when the cooling rate is increased from 5 °C/min to 40 °C/min, *T_c_* decreases from 215.1 °C to 202.2 °C. This is because rapid cooling shortens the time for molecular chain alignment, and sufficient power needs to be provided for crystallization by reducing the crystallization temperature (increasing undercooling).

From the DSC curve data, the crystallization peaks at different cooling rates are integrated and processed, and the relative crystallinity *X*(*T*) at a given temperature is obtained through Equation (6) [44]:

(6) $$X(T)=\frac{\int_{T_{0}}^{T}\left(dH_{c}/\mathrm{dT}\right)\mathrm{dT}}{\int_{T_{0}}^{T_{e}}\left(dH_{c}/\mathrm{dT}\right)\mathrm{dT}}$$

**Figure 4:**
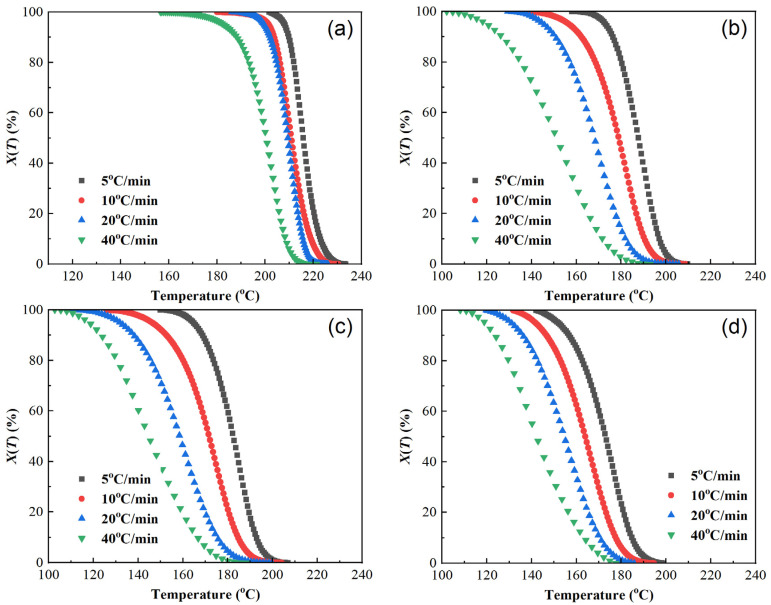
Plots of relative crystallinity versus temperature of samples at different cooling rates. (**a**) PET, (**b**) PEN-0.9%, (**c**) PEN-1.8%, and (**d**) PEN-9%.

Then, the temperature is converted into time data using Equation (7):

(7) $$t\;=\;\frac{T_{0}\;-\;T}{\varphi }$$

where *T* is temperature, °C; *T*_0_ and *T_e_* are the starting and ending temperatures of crystallization, °C; t is the crystallization time, min; and *φ* is the cooling rate, °C/min. The *X*(*T*)-*T* curves from Equation (6) are plotted in Figure 4.

As demonstrated in Figure 4, the *X*(*T*)-*T* curve trends of each sample are basically the same, showing an “inverse *S*” shape. The rate of change in *X*(*T*) is slower in the early and late stages of crystallization and faster in the middle stage. The excessively high cooling rate prevents the molecular chains from adjusting and folding in such a short period of time, resulting in crystallization hysteresis, which is also reflected in the figure. This manifests as the curve shifting to the left as the cooling rate increases, and the relative crystallinity at the same temperature decreases.

**Figure 5:**
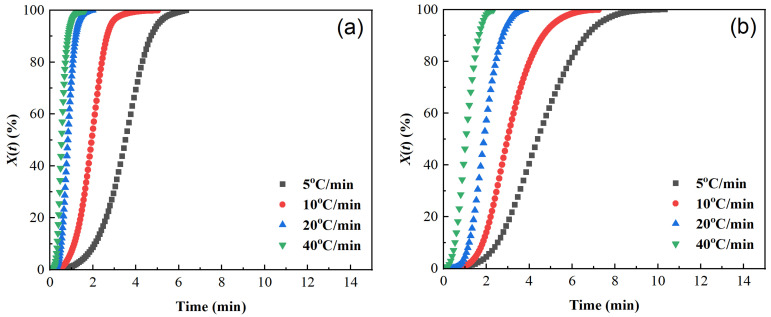

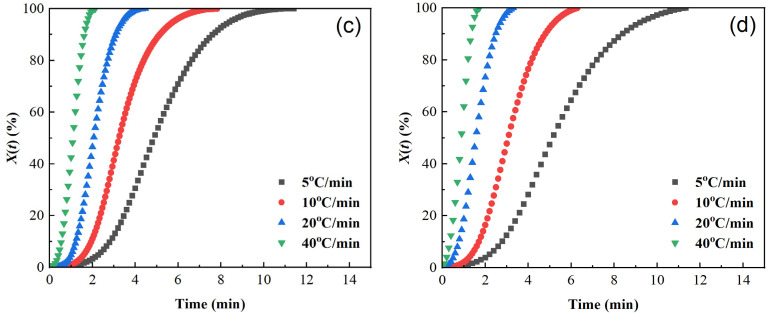
Plots of relative crystallinity versus time of samples cooled at different cooling rates. (**a**) PET, (**b**) PEN-0.9%, (**c**) PEN-1.8%, and (**d**) PEN-9%.

The curves of relative crystallinity *X*(*t*) versus crystallization time *t* of different samples are shown in Figure 5. The *X*(*t*) − *t* curves are all *S*-shaped, which corresponds to the three stages of the crystallization process [40,45,46]: the crystallization induction period, crystallization growth period, and crystallization completion period. The higher the cooling rate, the shorter the half crystallization time *t*_1/2_: the *t*_1/2_ of pure PET is reduced from 3.5 min at 5 °C/min to 0.54 min at 40 °C/min, indicating that rapid cooling can significantly accelerate the crystallization rate. At the same time, the area of the exothermic peak in Figure 3 decreases with increasing cooling rate. Although the time for the relative crystallinity to reach 100% is shortened in Figure 4, the final *X*(*t*) decreases, indicating that rapid cooling accelerates crystallization but leads to insufficient crystallization (an increase in amorphous parts).

The influence of PEN content on non-isothermal crystallization behavior can be analyzed by comparing the test results of different samples at the same cooling rate. The results showed that at the same cooling rate, an increase in PEN content had a significant inhibitory effect on crystallization behavior, and the inhibitory effect increased as the PEN content increased. The main manifestations are as follows:

(1) Decreased starting temperature *T*_0_ and peak temperature *T_c_* of crystallization: As shown in Figure 3 and Table 4, at a cooling rate of 10 °C/min, when the PEN content increases from 0% to 9%, T0 decreases from 230.3 °C to 194.5 °C and *T_c_* decreases from 211.1 °C to 166.5 °C, indicating that the introduction of PEN makes crystallization more difficult and reduces the temperature required to initiate crystallization.
(2) A slower crystallization rate and a reduced amount of crystallization: Figure 4 shows that at the same cooling rate, the higher the PEN content, the longer it takes for the relative crystallinity to reach 100%. As shown in Table 4, at 10 °C/min, the *t*_1/2_ (3.06 min) of PEN-9% was 57.7% longer than that of PET (1.94 min), and Δ*H_c_* (20.01 J/g) was 47.1% lower than that of pure PET (37.85 J/g), confirming that PEN hindered the migration and regular arrangement of PET molecular chains towards the crystal nucleus through the steric hindrance effect of the rigid naphthalene rings, resulting in a slower crystallization rate and a reduced amount of crystallization [47,48].

The above results indicate that the non-isothermal crystallization behavior of PET-PEN blends is influenced by both the cooling rate and PEN content. The cooling rate affects the crystallization kinetics by changing the degree of undercooling, while PEN alters the difficulty of crystallization, affecting the ability to crystallize, through a dual mechanism, characterized by “low-content heterogeneous nucleation, high-content steric hindrance”, and the steric hindrance effect becomes dominant with increasing PEN content.

### 3.5. Analysis of Non-Isothermal Cold Crystallization Behavior

**Figure 6:**
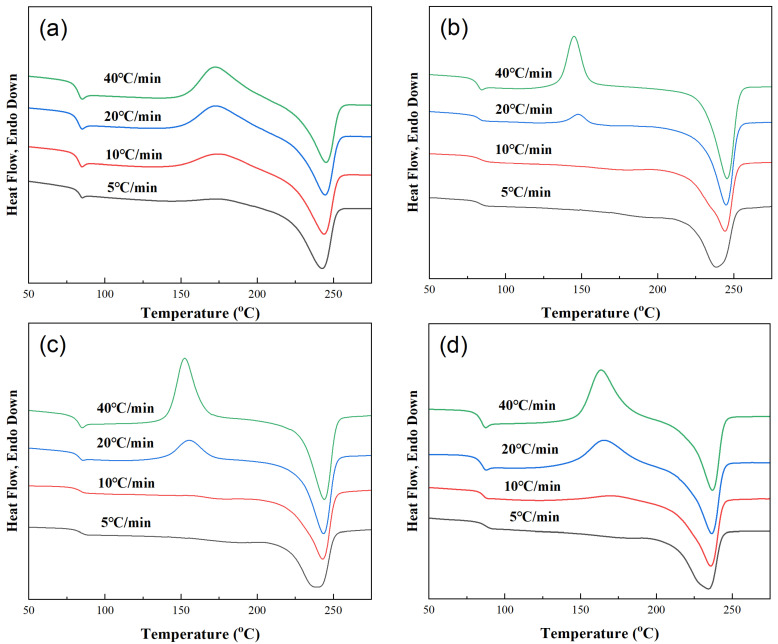
DSC heating curves obtained at a heating rate of 20 °C/min after samples were cooled at different cooling rates. (**a**) PET, (**b**) PEN-0.9%, (**c**) PEN-1.8% and (**d**) PEN-9%.

Figure 6 shows the DSC heating curves of the samples after they were cooled at different rates, followed by heating at a rate of 20 °C/min, reflecting the thermal transformation behavior of the crystallized sample. Quantitative parameters of the cold crystallization and melting processes were obtained from Figure 6 and included glass transition temperature *T_g_*, cold crystallization temperature *T_cc_*, enthalpy of cold crystallization Δ*H_cc_*, melting point *T_m_*, and melting enthalpy Δ*H_m_*, which are listed in Table 5.

From Figure 6 and Table 5, the following observations are made:

(1) Cold crystallization refers to the process in which the amorphous portion remaining after non-isothermal crystallization of the sample is arranged in a regular manner to form crystals during the heating process. Its pattern is closely related to the cooling rate and PEN content. The presence or absence of cold crystallization is related to the cooling rate.

The results show that pure PET exhibits cold crystallization peaks at all cooling rates, while samples PEN-0.9% and PEN-1.8% only exhibit cold crystallization peaks at high cooling rates (≥20 °C/min). PEN-9% exhibits no cold crystallization at 5 °C/min, while a cold crystallization peak with *T_cc_* = 170.59 °C appears at 10 °C/min. This is because at high cooling rates, the non-isothermal crystallization of the sample is less complete, and there are more residual amorphous parts, which need further heating for crystallization to occur. At low cooling rates, although PEN samples crystallize slowly, they can still fully crystallize and have fewer amorphous parts, so cold crystallization does not occur.

Analyzing the changes in the cold crystallization temperature *T_cc_* and cold crystallization enthalpy Δ*H_cc_* reveals that, for a fixed PEN content, the higher the cooling rate of the sample, the lower the cold crystallization temperature *T_cc_* and the higher the cold crystallization enthalpy Δ*H_cc_*, indicating that the amorphous portion remaining after rapid cooling is more likely to initiate crystallization at lower temperatures, and the sample has a higher amount of crystallization. At the same cooling rate, the higher the PEN content, the higher the cold crystallization temperature *T_c_*, indicating that the steric hindrance effect of PEN makes the movement of amorphous segments more difficult, and higher temperatures are required to initiate cold crystallization [49].

(2) In terms of melting behavior, the heating curves of all samples show a single melting peak, confirming a uniform crystal structure after crystallization. The pattern is described below.

For a fixed PEN content, increasing the cooling rate slightly increases the melting temperature *T_m_*. Due to the lower crystal regularity formed by rapid cooling, but more perfect crystals were formed during the cold crystallization process. At the same cooling rate, the higher the content of PEN, the lower the *T_m_*. This is because the embedding of PEN molecular chains into the PET lattice leads to an increase in crystal defects and a decrease in regularity.

The melting enthalpy Δ*H_m_* reflects the total amount of crystallization (including non-isothermal crystallization and cold crystallization). For the same PEN content in a sample, the higher the cooling rate, the closer Δ*H_m_* is to the theoretical complete crystallization enthalpy. This is because the amorphous portion remaining after rapid cooling is supplemented by cold crystallization, increasing the crystal yield. At the same cooling rate, higher PEN content causes a slightly lower amount of overall crystallization, but the amount of non-isothermal crystallization (Δ*H_c_* in Figure 3) significantly decreases, confirming that the inhibitory effect of PEN on the crystallization amount is mainly exerted in the non-isothermal crystallization stage.

### 3.6. Non-Isothermal Crystallization Kinetics

In this section, the non-isothermal crystallization kinetics of the samples are studied. Many classic methods can be used [50], such as the Lauritzen Hoffman method [51,52], Jeziory method, and Mo method [53,54,55]. These methods have been previously reported and applied to the material system in this study. The Jeziory method was used in this study on the basis of the previous literature [55].

#### 3.6.1. Jeziory Method

The Jeziory method, as a classic extension of the Avrami equation in non-isothermal crystallization systems [56], achieves quantitative extraction of kinetic parameters by introducing cooling rate correction.

The isothermal crystallization kinetics in the early stage of crystallization are commonly described by Avrami theory [57]:

(8) $$1\;-\;X_{t}=\exp\left(-Z_{t}t^{n}\right)$$

Taking the logarithm of both sides of the equation yields

(9) $$\mathrm{lg}\left[-\ln\left(1-X_{t}\right)\right]=\mathrm{lg}Z_{t}+n\mathrm{lg}t$$

In the equation, *X_t_* represents the crystallinity at time *t*; *Z_t_* represents the crystallization rate constant; and *n* represents the Avrami constant (mainly influenced by the nucleation mechanism and growth dimension).

Jeziormy extended Avrami theory to non-isothermal crystallization processes and corrected the *Z_t_* of the Avrami equation with the cooling rate [58]:

(10) $$\mathrm{lg}Z_{c}=\frac{\mathrm{lg}Z_{t}}{\Phi }$$

**Figure 7:**
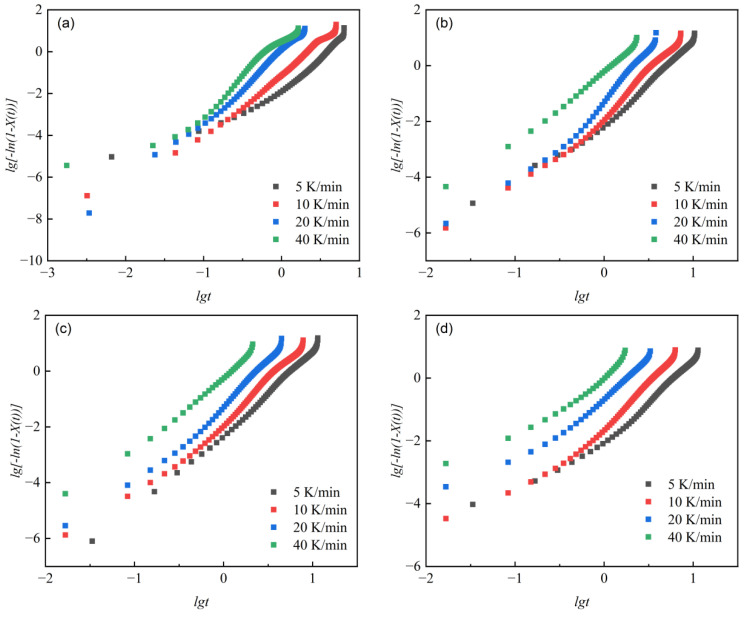
lg [−ln(1 − *X*(*t*))]-lg*t* relationship curves at different cooling rates *Φ*. (**a**) PET, (**b**) PEN-0.9%, (**c**) PEN-1.8%, and (**d**) PEN-9%.

In the equation, *Z_c_* is the corrected Jeziory crystallization rate constant; Φ is the cooling or heating rate, °C/min.

lg [−ln(1 − *X*(*t*))] versus *lgt* was plotted, as shown in Figure 7, to show their relationship during non-isothermal crystallization of different samples. We separately fitted the curves for the cases where crystallinity was above and below 90%. The non-isothermal crystallization kinetic parameters are shown in Table 6.

The change in the Avrami constant *n* directly reflects the evolution of the crystal nucleation mechanism and growth dimension [59]. When crystallinity was below 90%, it can be seen that the *n*_1_ value of pure PET is 2.496–3.272 (at different cooling rates), indicating that three-dimensional spherulite growth is dominant (nucleation time dimension 1 + growth space dimension 2). As the PEN content increases, the *n*_1_ value shows a decreasing trend: PEN-0.9% has *n*_1_ = 2.511–3.207, and PEN-9% has *n*_1_ = 1.771–2.495, indicating that the spatial hindrance of rigid naphthalene rings limits the spatial dimension of crystal growth, leading to a transition in growth mode towards two-dimensional lamellar crystals. It is worth noting that the *n*_1_ value of PEN-0.9% is slightly higher than that of PET at a cooling rate of 5 and 10 °C/min, which may be related to the transient enhancement of three-dimensional growth characteristics induced by the heterogeneous nucleation effect caused by low PEN content. The curve fitting results of relative crystallinity above 90% show that the Avrami constant *n* first decreases and then increases as the cooling rate increases. This is because, at high crystallinity, heterogeneous nucleation dominates at lower cooling rates, but the limited growth space leads to a decrease in the *n* value. However, at higher cooling rates, crystal growth is primarily governed by dispersed homogeneous nucleation, resulting in a recovery of the *n* value.

The variation law of the correction rate constant *Z_c_* reflects the regulation of crystallization rate: for the same sample, *Z_c_* increases with the increase in cooling rate, indicating that rapid cooling accelerates crystallization by increasing undercooling. At the same cooling rate, *Z_c_* decreases with increasing PEN content, and the *Z_c_*_1_ of PEN-9% decreases by about 31% compared to PET (at 10 °C/min), confirming that the steric hindrance effect of PEN significantly delays the crystallization process. However, the *Z_c_*_1_ of PEN-0.9% is slightly higher than that of PEN-1.8%, which once again confirms that the nucleation effect of low PEN content can partially offset the steric hindrance effect and is completely consistent with the abnormal pattern of variation in semi-crystallization time described in Section 2.4.

#### 3.6.2. Mo Method

The Mo method is an important method for studying non-isothermal crystallization kinetics; it overcomes the shortcomings of the Jeziory method in studying nucleation mechanisms and the unclear physical meanings of kinetic parameters, as well as the frequent occurrence of large linear fitting deviations and difficulty in obtaining reliable parameters in the Ozawa method [60]. For these reasons, the Mo method, as a new non-isothermal crystallization kinetics theory, has been widely applied in the analysis of polymers and composite materials.

Ozawa established the relationship between crystallinity and cooling rate at a given temperature and derived the non-isothermal crystallization kinetics equation [61]:

(11) $$1\;-\;X_{T}\;=\;\exp\left(-K\left(T\right)/\varphi^{m}\right)$$

Taking the logarithm of both sides of the equation yields

(12) $$\mathrm{lg}\left[-\ln\left(1-X(t\right)\right]=\mathrm{lg}\left[K\left(T\right)-m\mathrm{lg}\varphi \right]$$

where *X_T_* is the relative crystallinity at temperature *T*; *K*(*T*) is a function of temperature (related to factors such as nucleation mode, nucleation rate, and crystal growth rate) (°C/min^a−1^); and m is the Ozawa constant for the cooling or heating rate, °C/min.

Mo Zhishen et al. correlated Equation (9) of Jeziory’s method with Equation (12) of Ozawa’s method through the relationship between time and temperature (Equation (13)), obtaining Equation (14):

(13) $$t\;=\;\frac{\left|T\;-\;T_{0}\right|}{\varphi }$$

(14) $$\mathrm{lg}\varphi =\mathrm{lg}{\left[\frac{K(T)}{Z}\right]}^{1/m}-\frac{n}{m}\cdot \mathrm{lg}t$$

Let $F(T)\;=\;{\left(\frac{K(T)}{Z}\right)}^{1/m}$, $\alpha \;=\;\frac{n}{m}$; then,

(15) lgφ=lgF(T)−α·lgt

**Figure 8:**
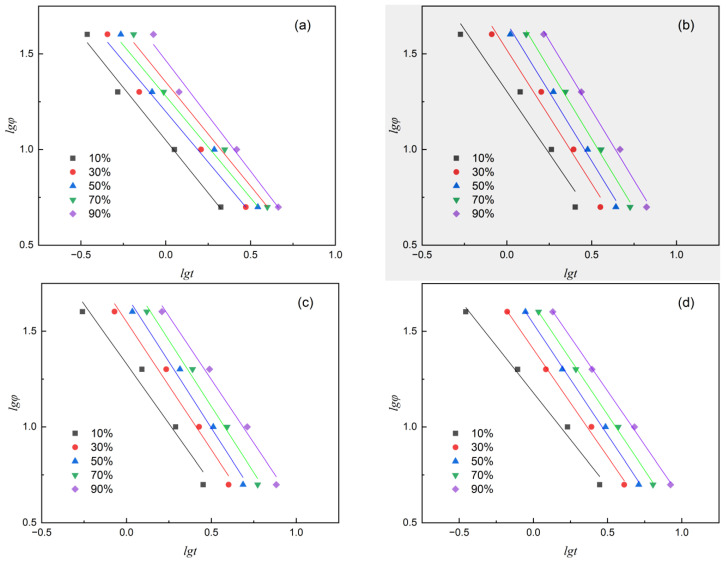
Plots of lg*φ* versus lg*t* of various samples at different relative crystallinity. (**a**) PET, (**b**) PEN-0.9%, (**c**) PEN-1.8% and (**d**) PEN-9%.

*F*(*T*) represents the characteristic cooling rate required to achieve a certain relative crystallinity, which characterizes the speed of the reaction crystallization rate. The larger the value of *F*(*T*), the higher the cooling rate required to achieve the same crystallinity. Non-isothermal kinetics are analyzed using Equation (15). Figure 8 is the result of plotting and fitting lg*φ* versus lg*t* at *X*(*t*) values of 10%, 30%, 50%, 70%, and 90%. The intercept lg*F*(*T*) and slope *α* were obtained through linear fitting, and the values are listed in Table 7.

Compared with the Jeziory method, the significant advantage of the Mo method is that it does not require the assumption of temperature dependence for nucleation and growth and maintains a high goodness of fit over a wide cooling rate range (5–40 °C/min). By correlating the *F*(*T*) value with the relative crystallinity temperature from the curve in Figure 5, it was found that *F*(*T*) is positively correlated with the slope of the curve, indicating that an increase in the characteristic cooling rate directly corresponds to an increase in kinetic resistance during the crystallization process.

The results in Figure 8 and Table 7 indicate that at the same relative crystallinity, *F*(*T*) shows a significant upward trend with increasing PEN content, but decreases when the PEN content is up to 9%. Indicating that when the PEN content is high, although the rigid segments limit the growth space of crystal nuclei, the increase in the number of crystal nuclei leads to an overall faster crystallization rate. This trend is also consistent with the pattern of t_1/2_ observed in Table 4. This change provides a quantitative basis for adjusting process parameters.

#### 3.6.3. Crystallization Activation Energy ΔE

Kissinger [62] proposed that the crystallization activation energy Δ*E* (kJ/mol) for non-isothermal crystallization can be calculated using Equation (16) [63] for the relationship between diameter and *T_c_*:

(16) $$\frac{d\left[\ln\left(\varphi /T_{c}^{2}\right)\right]}{d\left(1/T_{c}\right)}\;=\;-\frac{∆E}{R}$$

In the equation, *R* is the gas constant, with a value of 8.314 J/(mol K). Generally speaking, the Δ*E* of the non-isothermal crystallization of polymers consists of the free activation energy required for the regular arrangement of chain segments into the crystalline phase and the free activation energy required for the formation of critical crystal nuclei. The Δ*E* of each sample is obtained from the slope of the fitted curve of ln(*φ/T_c_*^2^) versus 1/*T_c_*, and the values are listed in Table 8.

**Figure 9:**
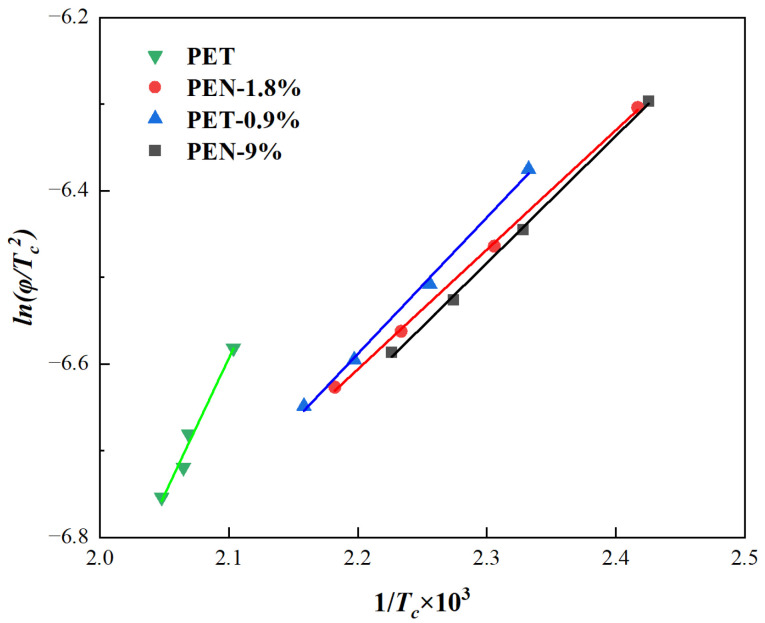
Plots of ln(*φ*/*T*_p_^2^) versus 1/*T_c_* of the samples.

From Figure 9 and Table 8, it can be seen that the Δ*E* of pure PET is −26.23 kJ/mol, which is much higher in absolute value than all PET-PEN blends. After adding PEN, the absolute values of Δ*E* in the blend decreased to below 14 kJ/mol, a decrease of over 49%: PEN-0.9% was −13.09 kJ/mol (a decrease of 49.9% compared to pure PET), PEN-1.8% was −11.48 kJ/mol (a decrease of 56.2% in absolute value), and PEN-9% was −12.21 kJ/mol (a decrease of 53.5% in absolute value).

Further analysis of the gradient effect of PEN content on Δ*E* reveals that as PEN content increases from 0.9% to 9%, the absolute value of Δ*E* shows a trend of “first decreasing, then slightly recovering and stabilizing”. It is worth noting that the absolute difference in Δ*E* between PEN-1.8% and PEN-9% is only 0.73 kJ/mol, indicating that when the PEN content exceeds 1.8%, its regulatory effect on Δ*E* is basically saturated [64].

The basis of the Δ*E* variation law is the macroscopic manifestation of the “steric hindrance effect” and “heterogeneous nucleation effect” of PEN molecular chains jointly regulating the PET crystallization process. Combined with the previous characterization results, the following can be further deduced:

(1) PEN reduces the energy barrier to crystallization mainly through the “dual effect” of steric hindrance.

According to FTIR, PET and PEN have good thermodynamic compatibility, and PEN molecular chains are uniformly dispersed in the PET matrix. The rigid naphthalene ring of PEN will cause steric hindrance. On the one hand, steric hindrance restricts the large-scale free migration of PET molecular chains; on the other hand, steric hindrance also “compresses” the space in which PET molecular chains can move, reducing irregular interactions between molecular chains. Under the “constraint” of PEN steric hindrance, the PET molecular chains that originally needed to overcome “large-scale intermolecular forces” to crystallize only need to complete a small-scale regular arrangement in local areas. Therefore, the energy barrier that needs to be overcome is significantly reduced (the absolute value of Δ*E* decreases), which also explains the observation that “the absolute value of Δ*E* of blends is lower than that of pure PET”.

(2) Low PEN content results in special physical behavior, and interference with heterogeneous nucleation is weakened.

The absolute value of Δ*E* of PEN-0.9% is slightly higher than those of PEN-1.8% and PEN-9%, which is related to the “heterogeneous nucleation effect” of low PEN content: at low content of PEN, its naphthalene ring induces the formation of heterogeneous crystal nuclei in PET, and the nucleation process requires a small amount of additional energy to stabilize the crystal nucleus structure, resulting in a slightly higher absolute value of Δ*E*. When the PEN content increases to 1.8% or above, the steric hindrance effect completely surpasses the heterogeneous nucleation effect, and the energy barrier further decreases and tends to stabilize. Therefore, the absolute value of Δ*E* no longer changes significantly with the increase in PEN content.

## 4. Conclusions

This study used PEN as a modifier to prepare PET-PEN blends with PEN contents of 0%, 0.9%, 1.8%, and 9%. FTIR, DSC, and other methods were used to systematically investigate the effects of the PEN content and cooling rate on the non-isothermal crystallization behavior, crystal structure, and kinetic properties of the blends and to clarify the regulatory effect of PEN on PET crystallization. The main research conclusions are as follows.

FTIR analysis confirmed that PET and PEN have excellent compatibility, and PEN is uniformly dispersed in the PET matrix.

The DSC results show that with an increase in the PEN content, the *T_g_* of the blend increases, while *T_m_* and relative crystallinity decrease. PEN hinders the regular arrangement of PET molecular chains through steric hindrance. The increase in cooling rate shifts the exothermic peak of crystallization towards lower temperatures and shortens *t*_1/2_, but it reduces the enthalpy of crystallization. In addition, PEN exerts a “dual effect” on *T_c_*: 0.9% PEN causes a sudden increase in *T_c_* due to heterogeneous nucleation, and when the content is ≥1.8%, steric hindrance leads to a decrease in *T_c_*. Pure PET exhibits cold crystallization peaks at various cooling rates, while samples with PEN did not exhibit cold crystallization at 5 °C/min. At the same rate, the higher the PEN content, the higher the *T_cc_*, and the higher the cooling rate of the same sample, the greater the Δ*H_cc_*.

The calculation results of crystallization kinetics using the Jeziory method show that PEN reduces the Avrami constant from 2.511–3.207 to 1.771–2.495 when crystallinity was below 90%, and the crystal growth dimension changes from three-dimensional to two-dimensional. *Z_c_* decreases with increasing PEN content. Mo method indicating a high goodness of fit and providing a basis for process optimization. The *F*(*T*) initially increases, but decreases when the PEN content reaches 9the %. The Kissinger method showed that PEN significantly reduced the absolute value of Δ*E*, and Δ*E* tended to stabilize when PEN content was ≥1.8%.

In summary, this study, through multiple analytical methods, found that PEN regulates PET non-isothermal crystallization through a mechanism characterized by “heterogeneous nucleation—diffusion control—steric hindrance effect”, providing theoretical support for the process design of PET-PEN blend films in high-end fields and serving as a reference for the study of non-isothermal crystallization of polyester materials.

## Figures and Tables

**Table 1:** Raw material ratio of PET/PEN blend.

Sample Name	PET (wt%)	PETN (wt%)
PET	100	0
PEN-0.9%	90	10
PEN-1.8%	80	20
PEN-9%	0	100

**Table 2:** Raw material properties of PET-PEN blends.

Sample	Intrinsic Viscosity (dL/g)	Carboxyl End-Group Content (mol/t)	Diethylene Glycol Content (%)
PET	0.798	11.9	0.63
PEN-0.9%	0.601	20.7	0.73
PEN-1.8%	0.671	15.4	0.60
PEN-9%	0.588	9.9	0.60

**Table 3:** DSC crystallization and melting parameters of samples obtained from Figure 2.

Sample	*T_g_* (°C)	*T_c_* (°C)	*T_m_* (°C)	*X_c_* (%)
PET	80.9	210.0	256.8	49.4
PEN-0.9%	81.7	189.1	248.1	32.1
PEN-1.8%	82.4	179.4	246.3	29.1
PEN-9%	84.5	173.6	239.2	25.5

**Table 4:** Non-isothermal crystallization parameters of samples.

Sample	*Φ* (°C/min)	*T*_0_ (°C)	*T_c_* (°C)	*T_e_* (°C)	Δ*H_c_* * (J/g)	*t*_1/2_ (min)
PET	5	233.4	215.1	201.7	39.9	3.52
	10	230.3	211.1	188.0	37.8	1.94
	20	226.0	210.1	186.1	29.7	0.83
	40	222.0	202.2	156.4	9.8	0.54
PEN-0.9%	5	209.7	190.2	158.0	32.6	4.39
	10	208.7	181.9	136.3	31.1	3.00
	20	205.6	170.1	129.4	26.0	1.88
	40	192.5	155.6	99.9	21.2	1.05
PEN-1.8%	5	206.7	185.1	149.9	31.1	4.86
	10	203.9	174.5	125.9	26.7	3.25
	20	199.6	160.5	110.4	19.1	2.07
	40	188.1	140.6	102.6	10.3	1.10
PEN-9%	5	198.6	176.0	142.0	28.0	5.13
	10	194.5	166.5	131.6	20.0	3.06
	20	185.9	156.3	119.9	6.9	1.57
	40	177.4	139.1	108.4	4.1	0.88

* Δ*H_c_* is experimental enthalpy obtained from DSC.

**Table 5:** Cold crystallization and melting behavior parameters obtained at a heating rate of 20 °C/min after samples were cooled at different cooling rates.

Sample	*Φ* (°C/min)	*T_g_* (°C)	*T_cc_* (°C)	Δ*H_cc_* * (J/g)	*T_m_* (°C)	Δ*H_m_* * (J/g)
PET	5	82.18	175.21	2.58	242.69	21.31
	10	81.42	172.88	6.87	244.01	23.03
	20	81.45	172.47	12.77	244.68	23.39
	40	81.38	172.58	17.55	245.34	24.74
PEN-0.9%	5	82.46	/	/	238.90	25.06
	10	83.65	/	/	244.51	28.73
	20	82.62	147.98	2.84	245.10	31.01
	40	80.67	145.02	13.34	245.70	31.36
PEN-1.8%	5	83.69	/	/	240.61	25.09
	10	83.08	/	/	243.22	26.75
	20	82.28	155.36	7.89	243.84	27.52
	40	81.26	152.41	20.17	244.14	30.04
PEN-9%	5	86.57	/	/	234.46	25.00
	10	83.37	170.59	1.97	235.77	30.04
	20	83.71	165.63	12.99	236.77	26.10
	40	83.14	163.34	21.22	237.10	26.65

* Δ*H_cc_* and Δ*H_m_* are experimental enthalpies obtained from DSC.

**Table 6:** Non-isothermal crystallization kinetic parameters of samples fitted using the Jeziormy method.

Sample	*Φ* (°/min)	*n*_1_	*Z_c_*_1_	*R* ^2^_1_	*n*_2_	*Z_c_*_2_	*R* ^2^_2_
PET	5	2.496	0.193	0.952	3.110	0.179	0.997
	10	2.982	0.343	0.989	1.858	0.682	0.989
	20	3.367	1.017	0.985	2.250	1.225	0.992
	40	3.272	1.741	0.951	1.879	1.628	0.994
PEN-0.9%	5	2.669	0.141	0.976	2.877	0.134	0.999
	10	2.997	0.184	0.978	2.627	0.245	0.990
	20	3.207	0.312	0.973	3.443	0.310	0.991
	40	2.511	0.794	0.996	3.214	0.699	0.989
PEN-1.8%	5	2.933	0.106	0.995	2.968	0.106	0.991
	10	2.960	0.171	0.985	2.648	0.171	0.987
	20	2.981	0.309	0.985	3.165	0.309	0.992
	40	2.548	0.773	0.995	3.728	0.773	0.992
PEN-9%	5	2.306	0.154	0.971	2.737	0.113	0.992
	10	2.495	0.236	0.973	3.076	0.175	0.995
	20	2.108	0.547	0.973	3.002	0.435	0.995
	40	1.771	0.944	0.974	3.868	0.859	0.993

**Table 7:** Non-isothermal crystallization kinetic parameters of samples calculated using the Mo method.

Sample	*X*(*t*), %	*F* (*T*)	*α*	*R* ^2^
PET	10	11.184	1.102	0.988
	30	15.787	1.056	0.987
	50	19.016	1.063	0.986
	70	22.561	1.088	0.985
	90	29.229	1.159	0.981
PEN-0.9%	10	20.098	1.297	0.953
	30	33.171	1.397	0.979
	50	45.783	1.443	0.991
	70	61.301	1.463	0.996
	90	86.191	1.462	0.994
PEN-1.8%	10	21.328	1.246	0.966
	30	35.406	1.336	0.981
	50	48.405	1.381	0.987
	70	62.589	1.379	0.991
	90	81.306	1.325	0.988
PEN-9%	10	15.089	0.978	0.989
	30	25.517	1.123	0.996
	50	34.623	1.163	0.998
	70	43.848	1.157	0.999
	90	56.418	1.128	0.999

**Table 8:** Kissinger crystallization activation energy of the samples.

Sample	Δ*E* (kJ/mol)
PET	−26.23
PEN-0.9%	−13.09
PEN-1.8%	−11.48
PEN-9%	−12.21

## Data Availability

The original contributions presented in this study are included in the article. Further inquiries can be directed to the corresponding author.
